# Revisiting pedicled latissimus dorsi flaps in head and neck reconstruction: contrasting shoulder morbidities across mysofascial flaps

**DOI:** 10.20517/2347-9264.2021.03

**Published:** 2021-02-25

**Authors:** Allen L. Feng, Hassan B. Nasser, Andrew J. Rosko, Keith A. Casper, Kelly M. Malloy, Chaz L. Stucken, Mark E. Prince, Steven B. Chinn, Matthew E. Spector

**Affiliations:** Department of Otolaryngology-Head and Neck Surgery, University of Michigan, Ann Arbor, MI 48109, USA.

**Keywords:** Pedicled latissimus dorsi flap, surgical flaps, myofascial flap, head and neck reconstruction

## Abstract

Free tissue transfer has become the gold standard for reconstruction within the head and neck. However, there are still many instances where pedicled locoregional flaps are the optimal reconstructive option. When myofascial tissue is needed, several options have been described throughout the literature. Various trapezius flaps have been used, although these have variable vascular anatomy and significant donor site morbidity. The pectoralis major myofascial flap has become a mainstay in head and neck reconstruction for its ease of harvest and reliability but suffers from similar issues with donor site morbidity. The pedicled latissimus dorsi flap (PLDF) is another reliable option that has been used for multiple different ablative sites within the head and neck. The thin, pliable structure of the latissimus dorsi makes it a viable option for many defects, and recent reports also support its feasibility for use in an interdisciplinary two-team approach. Furthermore, the donor site morbidity of the PLDF is minimal compared to other similar myofascial options. In this article, we describe the surgical considerations and operative techniques for PLDF transfer along with a review of its associated donor site morbidity.

## INTRODUCTION

Head and neck reconstruction has evolved tremendously over the last few decades. Pedicled locoregional flaps were the first workhorse flaps for reconstructing head and neck defects^[1–3]^, but have largely been replaced by free flaps. As our understanding of surgical and anatomic considerations improves, microvascular free tissue transfer has become the gold standard for reconstruction with success rates around 95%^[4–8]^. However, there are many situations where pedicled locoregional flaps still have merit. In patients at higher risk for thromboembolic events following microvascular anastomosis or those with vessel depleted necks, pedicled flaps present an appealing alternative to free tissue transfer. The low complications rate, need for a return to the operating room, and reduced operative time are important considerations in the right clinical context^[9–11]^.

When myofascial tissue is desired, the pectoralis major myofascial flap (PMMF) has been one of the most commonly used pedicled flaps since its original description in 1979^[1,2]^. Its robust tissue coverage, straightforward surgical technique, and reliability have all contributed to its widespread adoption^[12,13]^. Trapezius flaps were also described around that time, with the lower island trapezius flap (LITF) being first described in 1980^[14,15]^. Due to its location and reach, its use has been mostly described for reconstructing posterior cervical and occipital defects. Variable angiosomes have also brought into question its reliability, especially when harvesting with an overlying cutaneous paddle^[16–18]^. The upper trapezius flap (UTF) has also been described for tissue coverage within the neck or even intraoral defects^[19]^. However, its limited arc of rotation creates difficulties and typically requires a sacrifice of the spinal accessory nerve (CNXI).

The PMMF carries significant donor site morbidity associated with the harvest and loss of muscle function from the pectoralis major. Besides, the donor site morbidity associated with trapezius flaps and the need for intraoperative repositioning has prevented its more ubiquitous use. An alternative option for broad myofascial tissue coverage is the pedicled latissimus dorsi flap (PLDF). The first description of a pedicled myofasciocutaneous flap for head and neck reconstruction was given by Owens^[20]^ in 1955. In this study, Owens and the team described the use of a PLDF for reconstructing a mandibular defect. Since that time, the PLDF has been described throughout the literature for its utility in head and neck, chest wall, and breast reconstruction. It has also been used as an alternative to PMMF or trapezius flaps with improved donor site morbidity^[21–24]^. Previous concerns about patient positioning and its feasibility in an interdisciplinary two-teamed approach have limited its use in head and neck reconstruction, but recent descriptions have demonstrated the viability of a simultaneous harvest^[25,26]^.

The versatility of the PLDF, its minimal donor site morbidity, and its ability for simultaneous harvest make it a useful tool in head and neck reconstruction. Herein, we present a review of the clinical utility, surgical considerations, and associated morbidity for this flap in contrast with other commonly used myofascial flaps.

## PEDICLED LATISSIMUS DORSI FLAP

### Clinical utility

Free tissue transfer is the gold standard for complex reconstruction in the head and neck, but in cases where pedicled myofascial flaps are indicated, the PLDF is a robust and reliable option. The latissimus dorsi muscle provides broad tissue coverage and reaches most defect sites within the head and neck. The muscle itself is approximately 38 cm in length, 20 cm in width, and 0.8 cm thick^[27]^. Previous reports have demonstrated the PLDF’s ability to easily reach lateral temporal bone defects, orbito-cranial defects at the anterior and middle fossa, and posterior scalp defects^[28,29]^. The thin and pliable nature of the latissimus dorsi muscle makes it a viable candidate for these clinical situations in addition to the more common instances of re-surfacing required in the oral cavity, hypopharynx, or neck.

In particular, the proximity of the PLDF to the neck and hypopharynx makes it an excellent option for managing pharyngocutaneous fistulas or helping prevent complications after salvage laryngectomy. An overlying cutaneous skin paddle can also be easily incorporated with the latissimus dorsi muscle while still achieving primary closure of the donor site. This allows the PLDF to be used as a patch or interposition graft. Depending on the patient body habitus, the bulk of the overlying subcutaneous tissue on the latissimus dorsi muscle may create issues with circumferential reconstruction. However, the thin and pliable nature of the latissimus dorsi muscle itself makes it a reliable option as an interposition in most cases.

### Anatomic considerations

**Figure 1.**
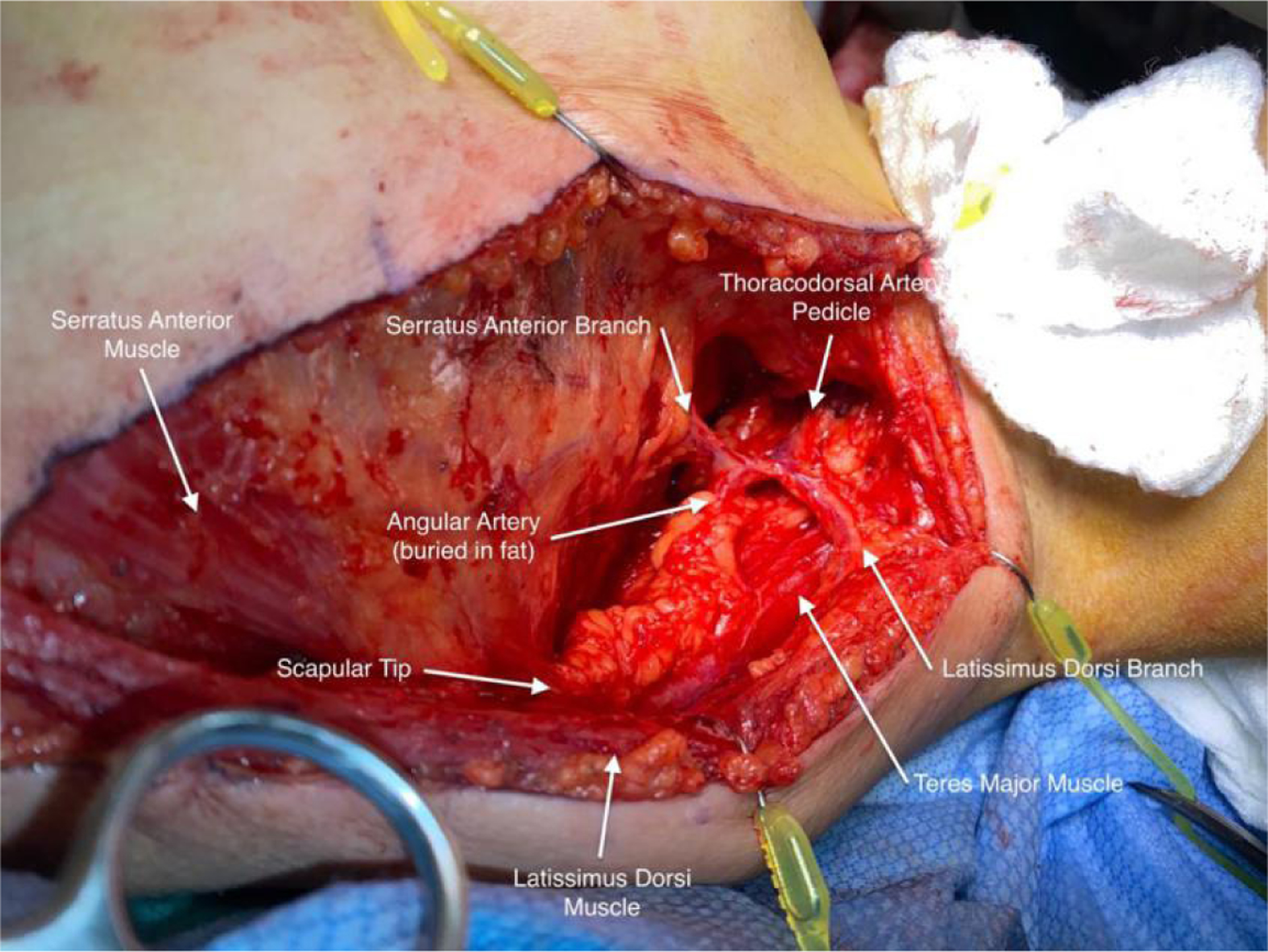
Representation of *in vivo* anatomy during the harvest of pedicled latissimus dorsi flap demonstrating vascular anatomy from the thoracodorsal system.

The latissimus dorsi is a broad muscle that originates from the inferior thoracic spinous processes, thoracolumbar fascia, iliac crest, and inferior ribs. It inserts on the inferior aspect of the intertubercular groove of the humerus through a thin tendon. Functionally it has been referred to as the back pec, serving to adduct the arm, largely assisting the teres major and pectoralis major muscles. The vascular supply to the latissimus dorsi muscle is classically described by Mathes and Nahai as a type V muscle, with a single dominant pedicle arising from the thoracodorsal system, and smaller segmental perforators from the posterior intercostal and lumbar arteries^[30]^. For the use of PLDF in head and neck reconstruction, the terminal latissimus dorsi branch of the thoracodorsal artery serves as the primary pedicle. Although there can be significant variability in the relationship between take-offs for the angular and serratus anterior branches from the thoracodorsal artery, the terminal latissimus dorsi branch reliably enters the deep surface of the muscle approximately 6 cm distal to the inferior scapular border^[31]^. In addition, the thoracodorsal artery can be dissected out of the latissimus muscle to create a longer pedicle and ease the arc of rotation. An *in vivo* depiction of this vascular anatomy is shown in Figure 1.

### Positioning

**Figure 2.**
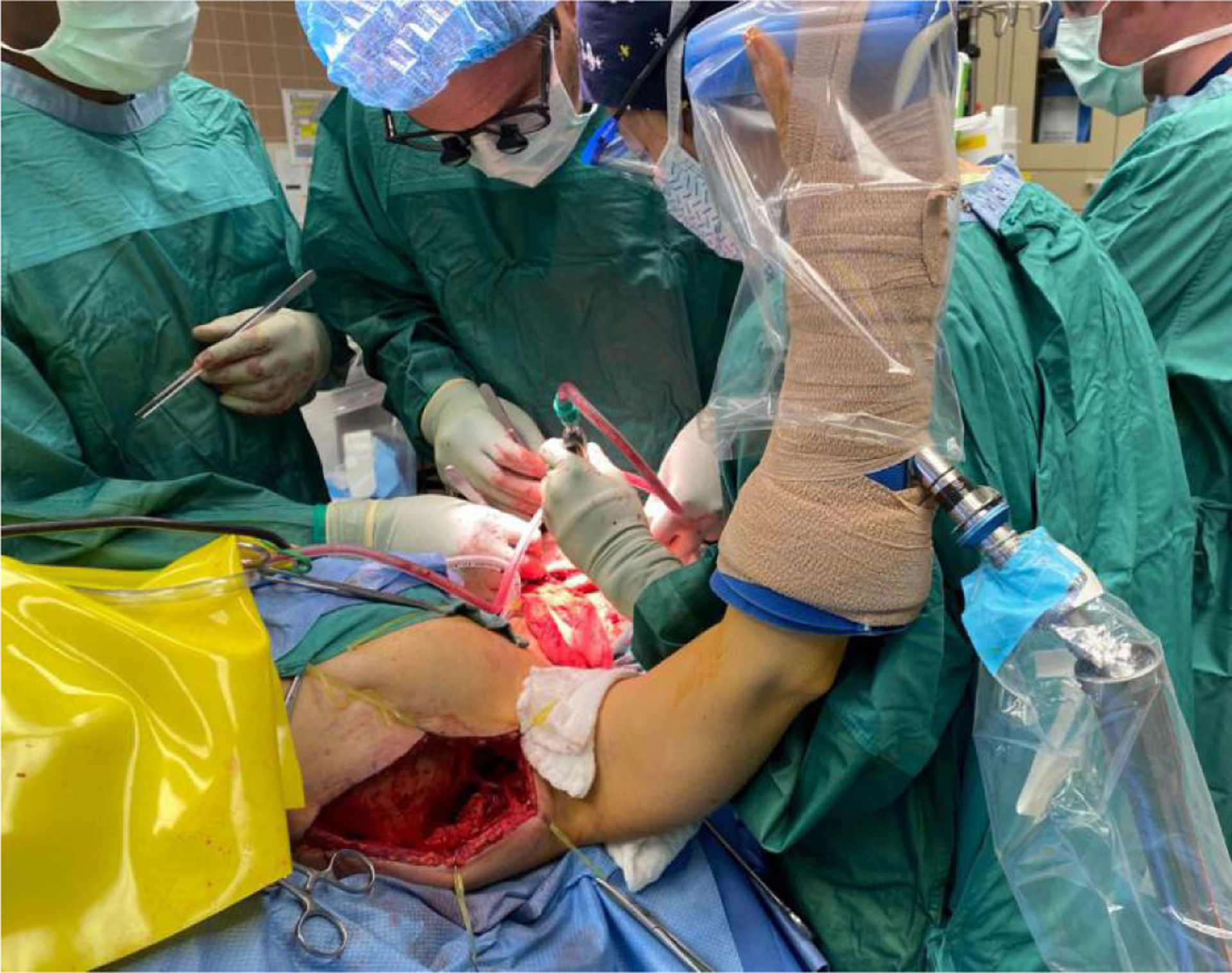
Demonstration of the two-team approach for the simultaneous harvest of pedicled latissimus dorsi flap and head and neck ablation using the Spider Arm Positioner system.

Traditionally, a harvest of a free or pedicled latissimus dorsi flap has been described through intraoperative patient re-positioning or sequential surgery^[21,28,32,33]^. However, more recent descriptions have used a supine position to eliminate the need for repositioning and allow a two-teamed approach^[34–36]^. The use of an upper extremity limb positioner (Spider Limb Positioner) to facilitate a simultaneous two teamed approach is described extensively by Stevens *et al.*^[25]^. This is achieved through positioning the patient on a bean bag in a semi-decubitus position while the Spider Limb Positioner holds the arm. This approach is depicted in Figure 2.

For smaller patients with reduced body habitus, a soft decubitus position with the arm extended to 90 degrees on an arm board can also be sufficient for PLDF harvest without the need for a Spider Arm. This is especially true when harvesting a myofascial flap alone. Incorporation of an overlying skin paddle may require brief retraction of the arm by an assistant to perform posterior cuts.

### Operative technique

With the arm in the proper position, the anterior border of the latissimus dorsi muscle can be easily palpated. This should approximate a line extending from the mid-axillary point down to a point between the anterior superior iliac spine and posterior superior iliac spine. The initial incision should be made at the inferior aspect of the anterior border of the latissimus dorsi muscle to prevent any inadvertent injury to the pedicle more superiorly. The inferior extent of the incision should be below the scapular tip and correlate with the length of the flap required to reach the primary defect. If a cutaneous skin paddle is harvested with the flap, careful skin paddle placement around the inferior aspect of the scapular tip is required to maximize the number of cutaneous perforators. After the initial incision, care is also taken not to shear the overlying subcutaneous tissue from the latissimus dorsi to preserve any musculocutaneous perforators.

**Figure 3.**
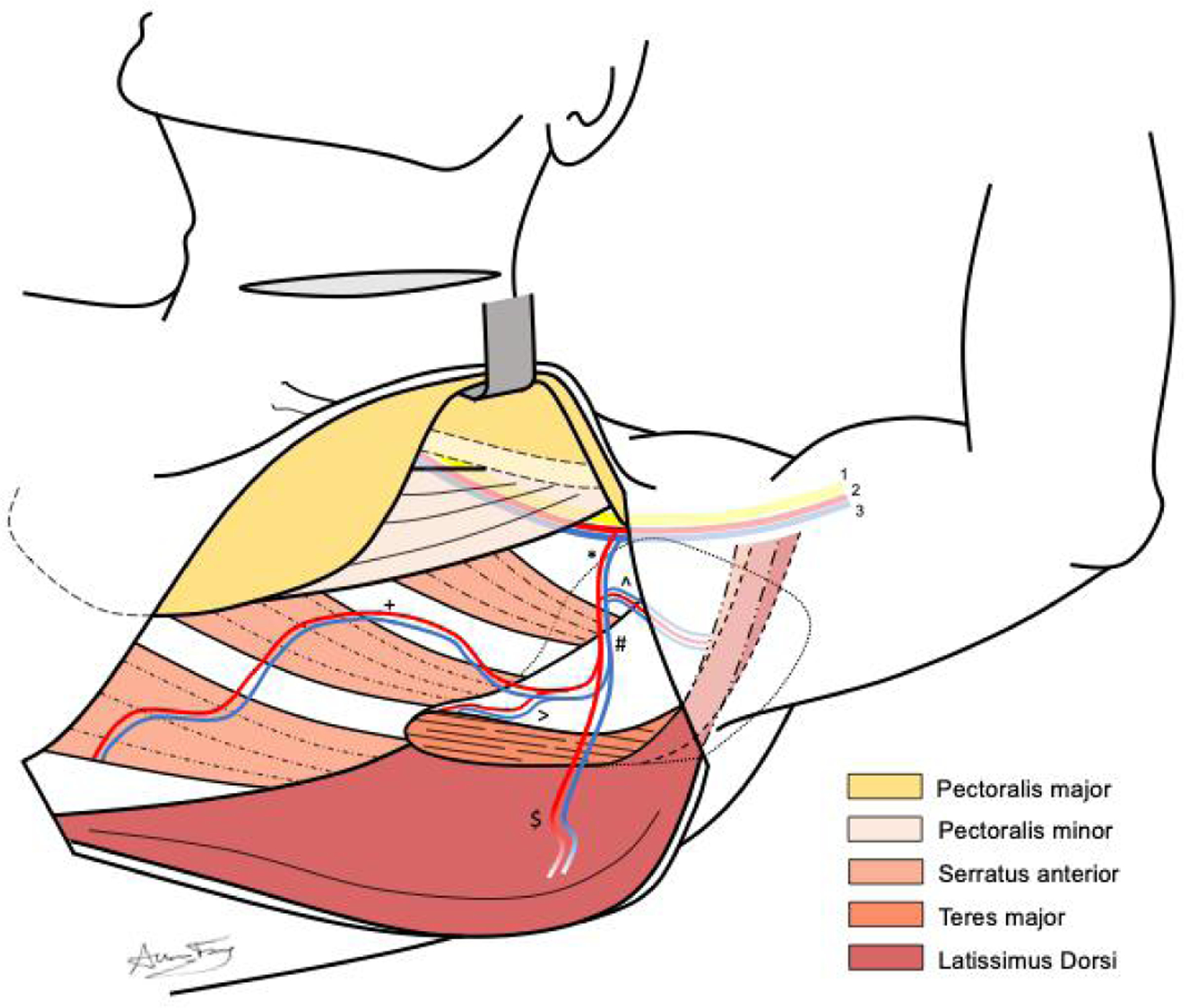
Anatomy of dissection for pedicled latissimus dorsi flap, demonstrating relevant muscular and neurovascular anatomy, including (*) subscapular, (^) circumflex scapular, (#) thoracodorsal, (>) angular, (+) serratus anterior, and ($) latissimus dorsi vessels. Also depicted are the brachial plexus (1), axillary artery (2), and axillary vein (3).

**Figure 4.**
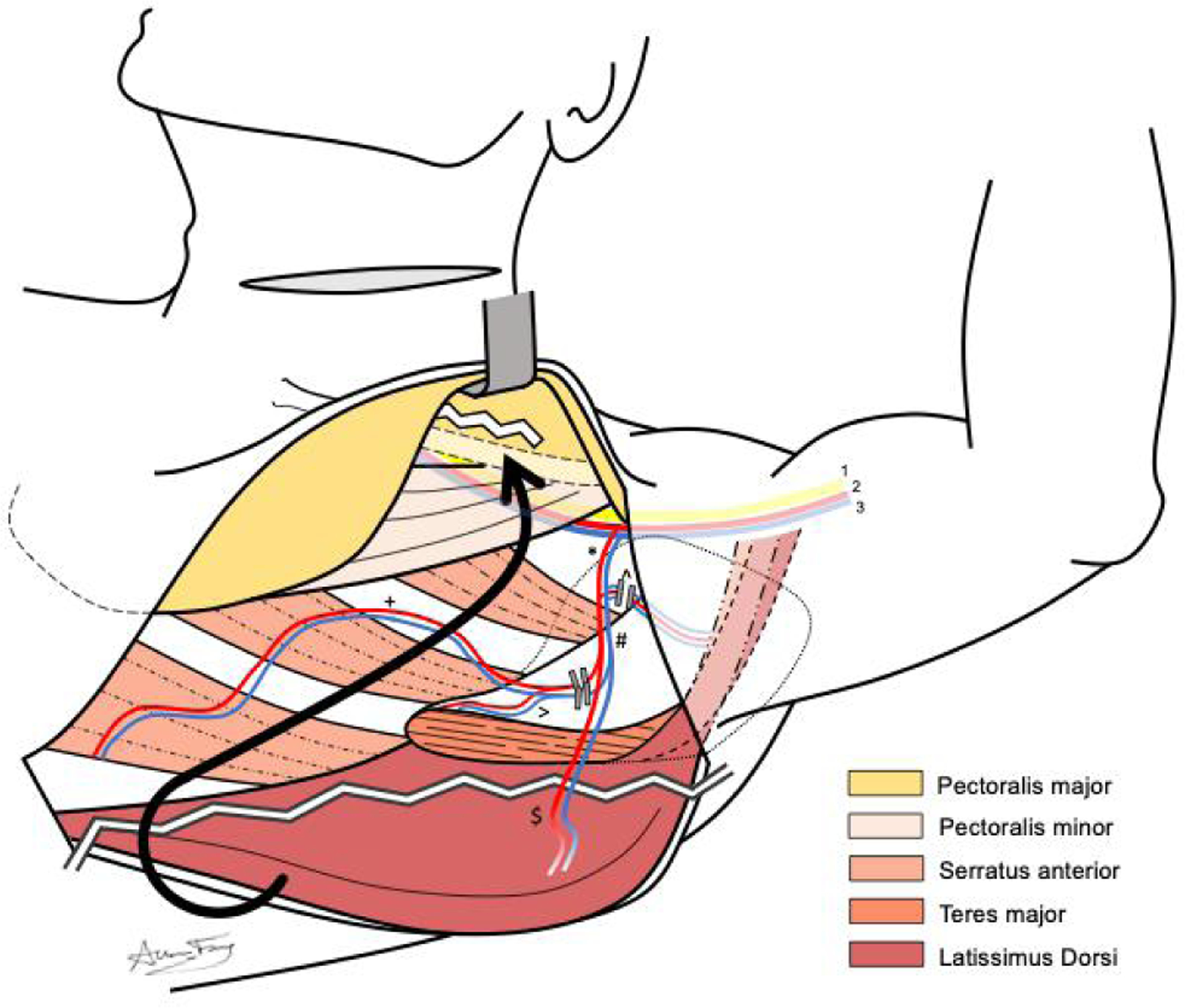
Anatomy of dissection for pedicled latissimus dorsi flap, demonstrating relevant muscular and neurovascular anatomy, including (*) subscapular, (^) circumflex scapular, (#) thoracodorsal, (>) angular, (+) serratus anterior, and ($) latissimus dorsi vessels. Also depicted are the brachial plexus (1), axillary artery (2), and axillary vein (3). The latissimus dorsi pedicle is isolated from other vascular branches, while the latissimus dorsi muscle is transected to free it both posteriorly and superiorly at the humeral attachment. The muscular flap is then tunneled superficial to the pectoralis major, over the clavicle and through the pectoralis major.

Once the anterior border of the latissimus dorsi muscle is visualized, an avascular plane can be easily developed between the deep surface of the latissimus dorsi muscle and the serratus anterior muscle of the chest wall. Once this plane has been confidently established, the incision can be extended superiorly towards the axilla. The layer between the deep latissimus dorsi muscle and anterior chest wall can then be bluntly dissected to reveal the scapular tip, teres major muscle, and vascular anatomy of the thoracodorsal system [Figure 1]. Depending on the patient body habitus, a variable amount of subcutaneous tissue should be bluntly dissected away from the vascular pedicle. Figure 3 depicts the anatomic view of this dissection. After identifying the latissimus dorsi branch, this segment of the muscle should be traced superiorly towards the subscapular system and its takeoff from the axillary artery and vein to maximize the PLDF arc of rotation. In our experience, it is important to take down the circumflex scapular veins to aid in pedicle rotation and confirm favorable geometry after rotation. The nerve to the latissimus dorsi will be intimately associated with the vascular pedicle and should be transected. Once the latissimus dorsi vascular pedicle has been confidently isolated, the angular and serratus anterior branches are ligated along with the circumflex scapular branch. Figure 4 demonstrates the ligation of these branches and subsequent muscular cuts used to isolate the latissimus dorsi from its inferior and humeral attachments.

**Figure 5.**
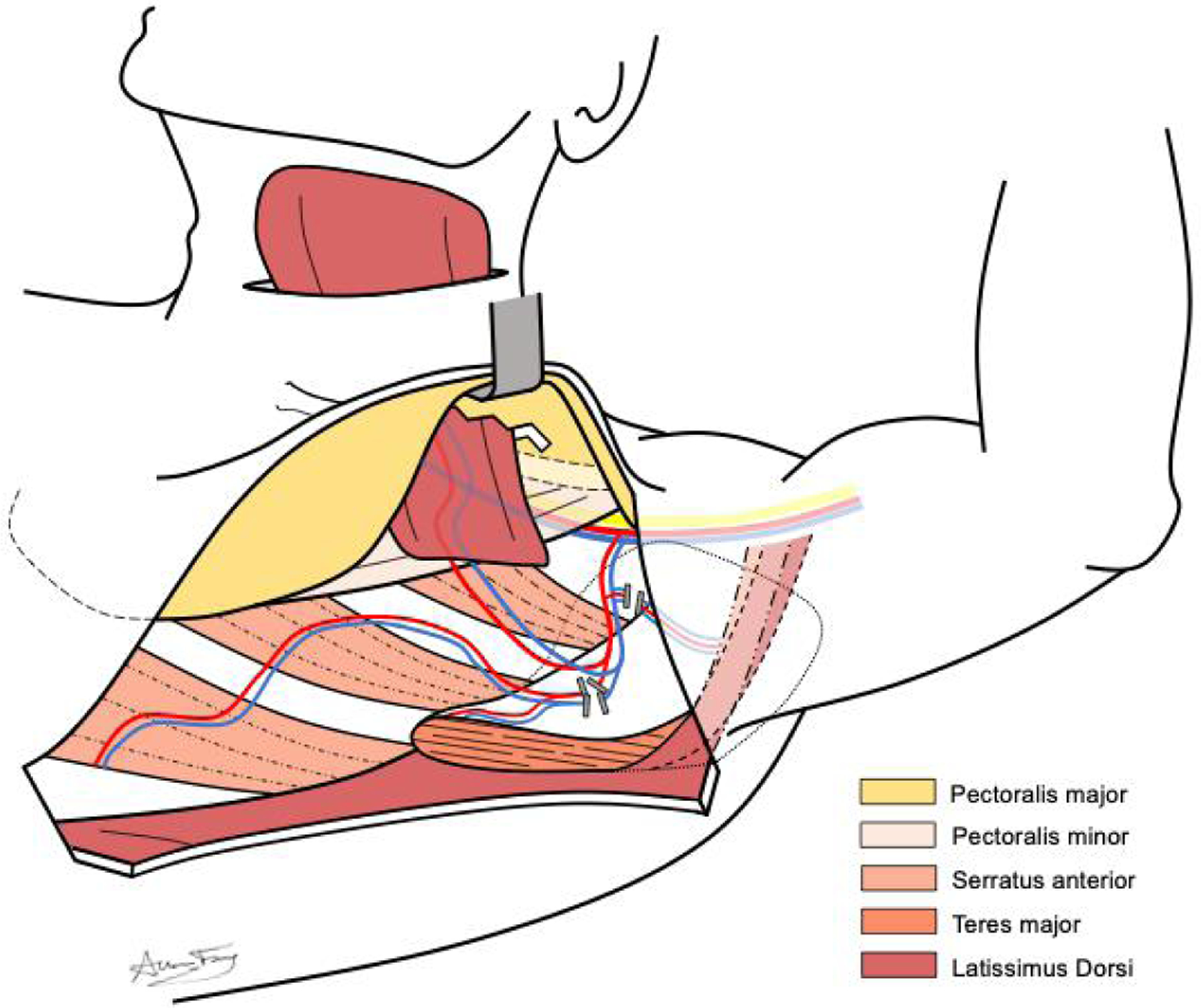
Anatomy of dissection for pedicled latissimus dorsi flap, demonstrating latissimus dorsi muscle flipped through a tunnel made in the pectoralis major muscle. Releasing the circumflex scapular pedicle allows for a significant arc of rotation.

Dissection is continued between the pectoralis major and minor muscles to create a tunnel through which the flap can pass to the neck. A cut is made through the attachment of the pectoralis major to the clavicle to connect the neck and chest tunnel. Once the vascular branches of the flap and muscular attachments are released, the flap can be passed through this opening as depicted in Figure 5.

## DISCUSSION

### Latissimus dorsi flap morbidity

The PLDF is a straightforward flap that can provide robust myofascial or myofasciocutaneous tissue coverage for multiple defects throughout the head and neck. Appropriate positioning and technique make a two-team approach possible, and the donor site morbidity of the PLDF is also favorable compared to other common pedicled myofascial donor sites. Multiple studies have investigated shoulder function following the sacrifice of the latissimus dorsi muscle^[23,24,37–39]^. Laitung and Peck describe one of the first objective assessments of shoulder function following the loss of the latissimus dorsi muscle as a free flap^[24]^. In that study, 13 of 19 patients had normal range of motion (ROM) in the affected arm, while the remaining 6 had some residual deficits in ROM (between 5° to 30°). Besides, 15 of 19 patients did not experience any subjective disability in their arm function. The maximum shoulder abduction power for each shoulder was also assessed; compared to a healthy control group, there was no significant difference in abduction power (kg) of the non-dominant (operated) arms. Furthermore, when comparing the non-dominant (operated) arm of each test subject to their own dominant (non-operated) arm, no difference was seen in abduction power (kg), subjective disability, or ROM.

These results are corroborated by more recent assessments of shoulder function following a latissimus transfer. Brumback *et al.*^[38]^ analyzed the shoulders of 17 patients who had undergone removal of a vascularized latissimus dorsi muscle. None of these patients reported any impediments in performing activities of daily living, nor needed any modifications in sports-related activities because of shoulder dysfunction. When compared to healthy controls, there was no objective difference in shoulder adduction, internal rotation, external rotation, or pushdown. Only when the arms were held in 60° of flexion, forced extension was weaker than in healthy controls. However, this was not accompanied by any loss in ROM. Fraulin *et al.*^[39]^ investigated the changes in muscle power and endurance for a group of 26 patients who had undergone pedicled or free latissimus muscle transfer. Fifteen of 26 had subjective difficulty with at least one activity since surgery, while only 4 of 26 had issues with a significant number of activities. The majority of these activities involved moving the arm above the head. Notable differences in power and endurance were seen for shoulder extension and adduction in females and males, however, patients did not see any additional deficits in work-simulated activities.

Other long-term studies also provide consistent findings in children who have had their latissimus dorsi muscle used as a myofascial flap. Osinga *et al*.^[40]^ describe a series of 3 patients in whom a myofascial latissimus dorsi flap was used within the first days after birth to cover a large myelomeningocele defect. After 8 years postoperatively, none of these patients experienced pain or shoulder restriction during normal daily activities. Objective measures for forward flexion, shoulder abduction, and external rotation were normal in each patient. Similarly, strength of abduction was not diminished on the operative side when compared to the non-operative side.

Despite the seemingly large biomechanical input from the latissimus dorsi, the objective and long-term shoulder dysfunction in patients receiving a latissimus dorsi muscle transfer is minimal. Weakness in shoulder extension or adduction is minimal, with deficits only seen in the form of increased muscle fatigue after extended use. This is largely due to the compensatory nature of the teres major - one of the most significant contributors for extension, adduction, internal and external rotation of the shoulder girdle^[37,41]^. Subsequent hypertrophy of this muscle after the loss of latissimus dorsi function can lead to a reduction in functional deficits and re-establishment of normal function.

### Trapezius and pectoralis major flaps: contrasting morbidities

Regarding trapezius flaps, both the LITF and UTF have been described for reconstructing a variety of defects within the head and neck, with a majority of instances occurring for posterior or lateral cranial defects. However, the associated morbidity for both flaps can be significant. For the UTF in particular, CNXI is typically sacrificed to increase the arc of rotation^[19]^. Although there is a paucity of objective analysis for shoulder and neck function following UTF, the resulting denervation of the trapezius and sternocleidomastoid muscles results in significant functional morbidity. Commonly referred to as “Shoulder Syndrome”, the resulting limitation in neck movement, accompanying atrophy, chronic shoulder pain, and reduced ROM is commonly seen after radical neck dissections^[42]^. This functional compromise has caused the UTF to fall out of favor. Studies have shown significant functional deficits in shoulder function, range of motion, and quality of life metrics for patients who underwent a sacrifice of CNXI during radical neck dissections compared to those who did not^[43]^. Similar functional deficits are seen with the LITF, although to a lesser degree. Both chronic shoulder pain and dysfunction are common minor complications following LITF^[18]^. Here, the proximal CNXI is not necessarily sacrificed, however, smaller branches are at risk. The variable angiosomes of the trapezius muscle has questioned the reliability of LITF. The transverse cervical artery (TCA) was thought to be the dominant pedicle to the trapezius; however, the dorsal scapular artery (DSA) provides a major contribution to the inferior aspect of the muscle. The relationship of the TCA and DSA is highly variable and several anatomic variations exist where each can be the dominant vascular supply to the trapezius^[16,17]^. This anatomic variability, significant donor site morbidity, and the need for intraoperative repositioning to a decubitus or prone position have limited the use of both UTF and LITF to very specific clinical situations.

In contrast, the PMMF is still widely used for its reliability, consistent anatomy, and ease of harvest. However, shoulder dysfunction following a PMMF harvest can be significant. Both objective and subjective assessments of shoulder function following PMMF are scarce throughout the literature, but consistent in their assessment of shoulder dysfunction. Sun *et al*.^[44]^ prospectively enrolled 46 patients undergoing PMMF and 46 matched control undergoing neck dissection only to assess changes in the Disability of the Arm, Shoulder and Hand (DASH) questionnaire at one year postoperatively. There was no significant difference in pre and postoperative DASH scores for the control group, while those undergoing PMMF saw a significant increase in postoperative DASH scores, nearly tripling their preoperative scores. Moukarbel *et al.*^[45]^ demonstrated similar results in a comprehensive assessment of objective and subjective shoulder dysfunction in patients undergoing PMMF. In their work, a case-control study of 8 patients undergoing total laryngectomy (TL), bilateral neck dissection (BND), and PMMF was compared to 10 patients undergoing TL and BND only. Objective analysis by a blinded physiotherapist demonstrated significant reductions in shoulder flexion angle and combined internal/external rotation angle for PMMF shoulders. A significant reduction in strength for shoulder flexion, external rotation, and adduction was also seen. Subjective assessments of shoulder function using the Shoulder Pain and Disability Index demonstrated a significantly higher disability score for those shoulders undergoing PMMF. Furthermore, physical analysis of the neck also demonstrated significant reductions in extension and total ROM on the ipsilateral side of PMMF. This was also confirmed on radiographic analysis, where total angular ROM was significantly reduced in the PMMF group when compared to controls.

**Figure 6.**
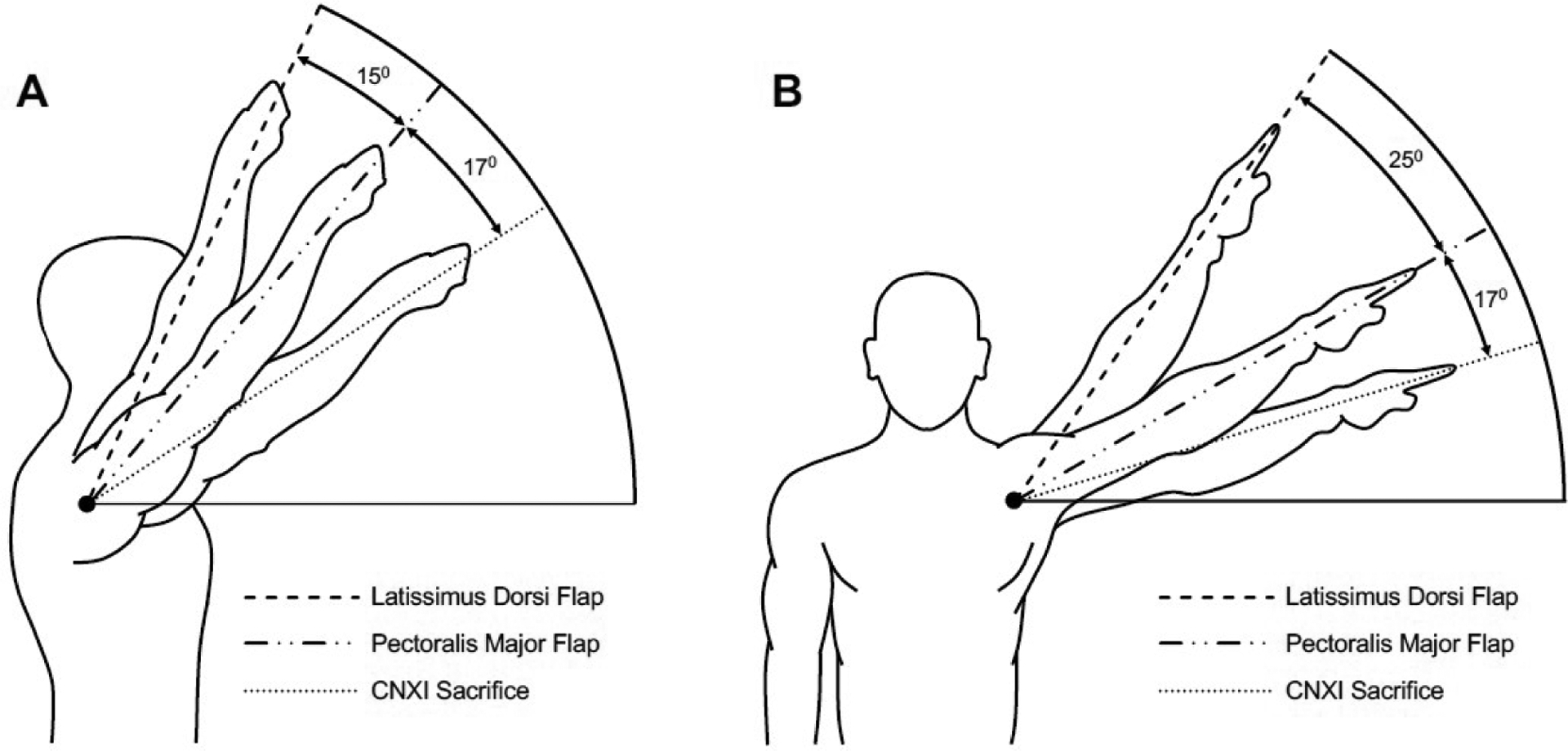
Relative deficits in passive shoulder flexion (A) and abduction (B) for latissimus dorsi donor site^[24]^, pectoralis major donor site^[45]^, and spinal accessory nerve (CNXI) sacrifice^[43]^. CNXI: spinal accessory nerve.

Although there is a lack of objective data to represent shoulder dysfunction associated with trapezius flap donor sites, the sacrifice of CNXI can be used as a surrogate for UTFs. Figure 6 demonstrates relative deficits in passive shoulder flexion [Figure 6A] and abduction [Figure 6B] between the latissimus dorsi donor site, pectoralis major donor site, and CNXI sacrifice. While this does not account for various factors like flap size or scar contracture, these comparisons show the expected passive ROM deficits associated with these myofascial flaps^[24,43,45]^. Overall, the current literature suggests reduced donor site morbidity for the PLDF when compared to other similar myofascial flaps, robust scientific evidence is needed to fully assess and compare these deficits. Direct comparisons for donor site function after PLDF, PMMF, LITF, or UTF will help inform the appropriate reconstructive option for a given defect.

## CONCLUSION

The PLDF is a reliable and easy to harvest myofasciocutaneous flap. Recent advances have made the simultaneous harvest and two-team approach viable options, obviating the surgical concerns that initially stigmatized the PLDF for head and neck reconstruction. In addition, the functional morbidity of this myofascial reconstructive is minimal in comparison to other commonly used myofascial pedicled flaps (PMMF, LITF, UTF).
